# Cellular senescence in skeletal muscle regeneration

**DOI:** 10.1186/s13619-026-00287-9

**Published:** 2026-05-07

**Authors:** Xingyuan Liu, Huating Wang

**Affiliations:** 1 Department of Chemical Pathology, Li Ka Shing Institute of Health Sciences, Chinese University of Hong Kong, Hong Kong SAR, China; 2 Department of Orthopaedics and Traumatology, Li Ka Shing Institute of Health Sciences, Chinese University of Hong Kong, Hong Kong SAR, China; 3 Center for Neuromusculoskeletal Restorative Medicine, Hong Kong Science Park, Hong Kong SAR, China

**Keywords:** Cellular senescence, Skeletal muscle regeneration, SASP

## Abstract

Skeletal muscle possesses a remarkable capacity for regeneration, driven by the activation and proliferation of Pax7-positive muscle stem cells within a dynamic niche that includes immune cells, fibro-adipogenic progenitors, endothelial cells, pericytes, and neural elements. Cellular senescence, a stress-induced program featuring stable cell-cycle arrest and the senescence-associated secretory phenotype (SASP), has emerged as a critical yet paradoxical regulator of this process. Accumulating evidence indicates that transient senescence, particularly in FAPs, macrophages, and other niche cells during acute muscle injury, plays a beneficial role in supporting muscle regeneration. These senescent cells promote cellular plasticity, enhance myoblast differentiation, facilitate phagocytic clearance of debris, and modulate inflammation and repair via timely SASP factor secretion. However, conflicting findings suggest that senescent cells exert detrimental effects, impairing regeneration by establishing a sustained pro-inflammatory and pro-fibrotic niche, especially when senescence persists in aged or dystrophic muscle. This review synthesizes the complex and contradictory roles of cellular senescence in skeletal muscle regeneration, underscores the distinction between transient pro-regenerative and persistent deleterious senescence, highlights the importance of cell-type-specific contributions, and emphasizes the need for precise characterization of senescent cell dynamics and fate. Resolving these discrepancies will be critical for developing targeted senotherapeutic strategies to enhance muscle regeneration in aging and degenerative diseases.

## Background

Skeletal muscle, constituting 30%–40% of human body mass, is essential for locomotion, metabolic regulation, and systemic homeostasis (Yamakawa et al. 2020). This tissue exhibits remarkable plasticity, capable of robust regeneration through a complex process. Regeneration of skeletal muscle primarily depends on the activation and proliferation of muscle stem cells (MuSCs), which repair damaged myofibers. In response to acute injury, the regeneration process follows a tightly coordinated sequence of phases. Initially, necrosis of damaged myofibers triggers an inflammatory response, characterized by the infiltration of immune cells that clear cellular debris and release cytokines. This inflammatory milieu is critical for activating quiescent MuSCs, prompting their exit from the niche, proliferation, and subsequent differentiation into myocytes. These myoblasts then fuse with each other or with surviving myofibers to regenerate and replace the damaged tissue. Additionally, the surrounding niche microenvironment, including immune cells, fibro-adipogenic progenitors, and endothelial cells, also contributes significantly to this process by providing essential signals for MuSCs regulation, extracellular matrix remodeling, and angiogenic support, ultimately leading to the restoration of functional muscle architecture (Yang and Hu 2018). In chronic muscle injury, like in Duchenne muscular dystrophy (DMD), dystrophic muscle tissue is trapped in a pathological cycle of continual degeneration and attempted regeneration. This process is driven by a pronounced and sustained inflammatory response. Although immune cell infiltration initially clears necrotic debris, the dystrophic environment causes this reparative inflammation to shift into a chronic, maladaptive state. Elevated pro-inflammatory factors create a toxic milieu that damages otherwise healthy fibers, impairs MuSC function, and promotes excessive fibrosis (Duan et al. 2021). Recent studies have increasingly focused on cellular senescence, a stress-induced response program, in the context of skeletal muscle regeneration (Dungan et al. 2022; Moiseeva et al. 2023; Young et al. 2022). Senescent cells accumulate with aging and age-associated diseases, and are believed to drive tissue aging primarily by establishing a chronic inflammatory and fibrotic microenvironment (Baker et al. 2016; Chang et al. 2016; Xu et al. 2018). Interestingly, senescence is generally thought to play a beneficial role in tissue regenerative process (Antelo-Iglesias et al. 2021). Effective muscle regeneration may depend on the transient local accumulation of senescent cells and cellular interplay by their secretion of the senescence-associated secretory phenotype (SASP) factors (Henrot et al. 2023). This review focuses on the complex and paradoxical roles of cellular senescence in skeletal muscle regeneration, highlighting the heterogeneity of senescent cells and necessitating cell-type-dependent elucidation.

## Skeletal muscle regeneration: stem cell and niche microenvironment

Skeletal muscle regeneration is a highly coordinated process involving the interplay among MuSCs and multiple cell types within a microenvironment known as the stem cell niche. This niche orchestrates tissue repair after injury, maintaining homeostasis through dynamic cellular crosstalk.

### MuSCs

MuSCs serve as the central drivers of skeletal muscle regeneration. Normally, they reside between the basal lamina and sarcolemma, maintaining a quiescent state via PAX7-dependent transcription and cell-cycle inhibitors, called quiescent muscle stem cells (QSCs) (Li et al. 2025a). Upon extrinsic stimuli, such as injury and exercise, MuSCs are activated to enter the cell-cycle and upregulate MYOD expression (Tedesco et al. 2010). Then, activated MuSCs proliferate as myoblasts and differentiate into myocytes characterized by MYOG expression, which subsequently fuse to form nascent myotubes and new myofiber to repair the damaged myofiber eventually (Feng et al. 2024; Yu et al. 2021). During proliferative phase, a subset of activated MuSCs undergoes self-renewal, dividing asymmetrically and returning to quiescent state under the basal lamina to replenish the MuSC pool (Byun et al. 2024).

### Immune cells

Under normal physiological conditions, immune cells continuously circulate within the blood and the lymphatic system. However, following muscle injury, supplementary leukocytes swiftly infiltrate muscle tissue and release cytokines and growth factors that modulate the local microenvironment, orchestrating the repair process (Saini et al. 2016). During the initial inflammatory phase, neutrophils rapidly infiltrate the damaged tissue within hours, clearing cellular debris via phagocytosis and secreting cytokines and chemokines that recruit macrophages (Ziemkiewicz et al. 2021). Concurrently, the recruited pro-inflammatory (M1) polarized macrophages, along with neutrophils, phagocytose necrotic material and secrete growth factors, cytokines, inflammatory mediators that activate MuSCs and promote myoblast proliferation (Kharraz et al. 2013). In the regenerative phase, a critical shift occurs toward anti-inflammatory (M2) macrophage polarization, influenced in part by regulatory T cells (Tregs) through the secretion of IL-10, IL-13, and IL-4 (Burzyn et al. 2013a). This M1-to-M2 transition is essential for effective muscle regeneration: M1 macrophages initiate the inflammatory response and promote MuSCs activation and proliferation, whereas M2 macrophages resolve inflammation and support both continued proliferation and terminal differentiation and fusion of MuSCs (Chazaud 2020; Patsalos et al. 2021). Treg populations markedly expand during acute muscle injury(Burzyn et al. 2013b). Besides mediating macrophage polarization, they secrete amphiregulin, directly enhancing MuSC proliferation and differentiation (Burzyn et al. 2013a). Finally, during the remodeling phase, M2 macrophages and Tregs facilitate extracellular matrix (ECM) reorganization and inflammation resolution (Bernard et al. 2022; Fang et al. 2021).

### Fibro adipogenic progenitors

Fibro-adipogenic progenitors (FAPs)—resident mesenchymal stromal cells marked by PDGFRα⁺ expression—are indispensable regulators of skeletal muscle regeneration. Upon acute injury, FAPs rapidly expand and secrete growth factors like WISP1 and IGF-1 that directly activate MuSCs, promoting their proliferation and differentiation into myofibers (Ancel et al. 2019; Luo et al. 2025). Activation of IL-4/IL-13 signaling promotes proliferation of FAPs to support myogenesis while inhibiting their differentiation into adipocytes. In addition, FAPs are also involved in phagocytosis of necrotic debris and produce ECM components that structurally support repair (Heredia et al. 2013). While FAPs are crucial for effective regeneration, their dysregulation, particularly persistent activation and failure of apoptotic clearance, can also drive pathological outcomes. Serving as the primary source of intramuscular adipocytes, FAPs promote adipogenesis and fat infiltration (IMAT/myosteatosis), particularly when IL-4/IL-13-mediated inhibition is lost or fibrogenic/adipogenic cues predominate. This dual fibro-adipogenic potential underlies fibrosis and fatty degeneration in diseases such as muscular dystrophy, aging, and metabolic disorders (Flores-Opazo et al. 2024; Loomis and Smith 2023).

### Other niche supporting cells

Beyond MuSCs, immune cells, and FAPs, endothelial cells (ECs) critically orchestrate muscle regeneration by promoting angiogenesis and maintaining MuSC quiescence. ECs secrete the Notch ligand Dll4, which activates Notch signaling in MuSCs to preserve their quiescent state. In turn, MuSCs produce VEGF-A to stimulate ECs for vascular growth, thereby enhancing nutrient delivery to regenerating areas (Koike et al. 2022). EC-derived angiocrine factors also support myoblast differentiation and fusion into nascent myotubes (Li et al. 2025a). Pericytes (PCs), localized adjacent to ECs, stabilize nascent vasculature and secrete IGF-1 to promote myoblast differentiation and myofiber maturation directly (Rodríguez et al. 2024). Neural cells facilitate functional recovery by re-establishing neuromuscular junctions (NMJs). Motor neurons release agrin and neuroregulin-1, which cluster acetylcholine receptors (AChRs) on regenerated myofibers, restoring electromechanical coupling (Yadav and Dabur 2024).

## Cellular senescence

Cellular senescence is a state of stable and irreversible cell cycle arrest induced by diverse stressors, including telomere dysfunction, DNA damage, oncogene activation, oxidative stress, and mitochondrial impairment (Di Micco et al. 2021). First described by Hayflick and Moorhead in 1961, it was initially observed as replicative senescence in human fibroblasts, which ceased proliferation after approximately 50 divisions—a phenomenon termed the "Hayflick limit" (Hayflick and Moorhead 1961). Unlike quiescence and terminal differentiation, which are governed by developmental programs, senescence primarily arises from stress-induced responses (Herranz and Gil 2018)—particularly relevant in the context of skeletal muscle injury and regeneration.

### Defining features of senescent cells

Senescent cells represent a stable and essentially irreversible arrest of the cell cycle, typically orchestrated through activation of the p53/p21 and p16^INK4a^/pRB tumor suppressor pathways, which converge to inhibit cyclin-dependent kinase activity and prevent retinoblastoma protein phosphorylation, thereby enforcing durable proliferation cessation (Lucas et al. 2023; Suryadevara et al. 2024; Wan et al. 2021). This growth arrest is accompanied by the acquisition of a senescence-associated secretory phenotype (SASP), characterized by the sustained release of proinflammatory cytokines such as interleukin-6 (IL-6) and interleukin-8 (IL-8), chemokines including CCL2 and CCL7, growth factors such as transforming growth factor-β (TGF-β), and extracellular matrix–modifying enzymes like matrix metalloproteinases (MMPs) and their endogenous inhibitors (TIMPs). The SASP is transcriptionally regulated by key signaling nodes, including NF-κB, p38 mitogen-activated protein kinase (p38MAPK), mammalian target of rapamycin (mTOR), CCAAT/enhancer-binding protein β (C/EBPβ) and AP-1 family transcription factors (Birch and Gil 2020; Martínez-Zamudio et al. 2020; Saito et al. 2024; Schafer et al. 2020). Morphologically, senescent cells exhibit pronounced hypertrophy and flattening, increased lysosomal biogenesis evidenced by elevated senescence-associated β-galactosidase (SA-β-Gal) activity at pH 6.0, depletion of nuclear lamin B1, accumulation of autofluorescent lipofuscin granules, and persistence of DNA damage foci such as γH2AX-positive DNA segments with chromatin alterations reinforcing senescence-associated chromatin structures (DNA-SCARS) (López-Otín et al. 2023; Zeng et al. 2024). Additionally, senescent cells display upregulated expression of specific plasma membrane proteins, including dipeptidyl peptidase-4 (DPP4), TNF receptor superfamily member 10D (TNFRSF10D), NOTCH1, NOTCH3, CD36, oxidized vimentin, intercellular adhesion molecule-1 (ICAM1), and the urokinase-type plasminogen activator receptor (uPAR), which serve as phenotypic markers for their identification (Rossi and Abdelmohsen 2021). Collectively, these molecular, secretory, and structural alterations not only demarcate the senescent phenotype but also underpin its dual roles in tissue remodeling and in promoting chronic inflammation and age-associated pathologies.

### The functions of cellular senescence in development and tissue regeneration

Cellular senescence plays multifaceted and context-dependent roles across various biological processes. Initially characterized as a tumor-suppressive mechanism that prevents the proliferation of damaged or potentially malignant cells (Ajoolabady et al. 2025), it is now evident that chronic senescence is a key driver of aging and age-related diseases, as senescent cells progressively accumulate in aged organisms due to impaired immune clearance and persistent stress‑response pathways and the sustained SASP fosters chronic inflammation, fibrosis, and stem cell dysfunction, ultimately impairing repair and promoting tissue degeneration (Antelo-Iglesias et al. 2021). The detrimental role of cellular senescence has been summarized in many excellent reviews, such as those by Huang et al. (2022) and Li and Wang (2026).

Although often associated with aging and chronic inflammation, senescent cells can exert transient, beneficial effects in development, tissue repair and regeneration when promptly cleared after fulfilling their role (Lavarti et al. 2025). For example, during mammalian embryonic development, transient senescence occurs in a programmed manner at specific sites, where p21-dependent senescent cells display SA-β-Gal activity and secrete SASP factors such as FGF ligands to guide tissue patterning and morphogenesis via paracrine signaling. These senescent cells are subsequently cleared by macrophage-mediated apoptosis, enabling tissue remodeling without persistent inflammation. Disruption of this process in p21-deficient mice leads to developmental abnormalities, underscoring its essential, non-redundant role in normal development. In cutaneous tissue, transient senescence in fibroblasts and ECs has been shown to promote wound healing by facilitating myofibroblast differentiation and angiogenesis via SASP-mediated secretion of PDGF-AA and VEGF. This role is supported by evidence that selective elimination of p16^INK4a^-positive cells through *p16-3MR* (Trimodality Reporter) mice delays wound closure, which was rescued by exogenous PDGF-AA treatment (Demaria et al. 2014). Furthermore, induction of senescence by the matricellular protein cellular communication network factor 1 (CCN1) via DNA damage response acts as an antifibrotic mechanism during wound repair. CCN1 deficiency leads to excessive fibrosis, which can be reversed through topical CCN1 application that reinstates senescence and attenuates fibrotic progression (Jun and Lau 2010). Similar mechanisms have been observed in other tissues. In the liver, CCN1-induced senescent stellate cells secrete matrix metalloproteinases that promote tissue remodeling and inhibit fibrosis, while in the heart, both CCN1 and Arg1-driven transient senescence in fibroblasts support regeneration by enhancing cardiomyocyte proliferation, reducing fibrosis, and secreting reparative factors such as Cathepsin S via the SASP (Feng et al. 2019; Kim et al. 2013; Krizhanovsky et al. 2008; Sarig et al. 2019; Zhang et al. 2024). These regenerative effects are critically dependent on the transient nature of the senescent state; prolonged persistence of senescent cells shifts the SASP toward a pro-inflammatory and pro-fibrotic profile, ultimately impairing tissue repair. Thus, understanding the temporal and tissue-specific dynamics of senescence is essential for developing therapeutic strategies that harness its regenerative potential while minimizing long-term pathological consequences.

## Senescent cells in skeletal muscle regeneration

With advancing insights into the diverse roles of cellular senescence in tissue repair, this process has been increasingly recognized as a key regulatory process in muscle regeneration. Previous studies have demonstrated that cellular senescence is a defining feature of age-related skeletal muscle disorders, and that targeted elimination of senescent cells can mitigate tissue damage while enhancing muscle strength in aged mice (Kedlian et al. 2024; Li et al. 2025b; Walter et al. 2024; Zhang et al. 2022). However, consensus has yet to be reached regarding the specific roles of cellular senescence in the regeneration of skeletal muscle (Table 1).

**Table 1:** Studies of senescent cells in muscle regeneration

**Author (Year)**	**Animal Age**	**Senescent Cell Type**	**Appearance time**	**Key Evidence**	**Function in Regeneration**	**Method**	**Mechanism**
Le Roux et al. (2015)	8 weeks	ECs, Macrophages	10 and 20 dpi	SA-β-Gal↑, Ki67↓	Potential pro-regenerative	N.A.	Not identified
Chiche et al. (2017)	6–8 weeks	Not identified	10 dpi	SA-β-Gal↑, p16^INK4a^↑, p19^Arf^↑, SASP↑	Pro-regenerative	Senescent cell clearance with ABT-263 or in *p16-3MR* mice reduced IL-6 and cell plasticity	Senescent cells promote in vivo reprogramming & cellular plasticity via SASP factors (e.g., IL-6)
Saito et al. (2020)	8–10 weeks	FAPs	4 dpi	SA-β-Gal↑, p53↑, p16^INK4a^↑, p19^Arf^↑, p21↑, γH2AX↑	Pro-regenerative	Transplantation of p53(+/+) senescent FAPs promoted regeneration	Senescent FAPs create a state of regenerative inflammation by SASP expression and induce apoptosis to prevent fibrosis
Dungan et al. (2021)	3 months	CD11b + immune cells	7 and 14 dpi	SA-β-Gal↑	Pro-regenerative	Senescent cell clearance with D + Q enhanced regeneration in old mice but impaired regeneration in young mice	Not identified
	20 months				Anti-regenerative		
Young et al. (2022)	8 weeks	FAPs, Macrophages, ECs, Tenocytes, MuSCs	2 and 5 dpi	p53↑, p57^Kip2^↑, p27↑, p21↑, GLB1↑	Pro-regenerative	Senescent cell clearance with ABT-263 impaired muscle repair	Not identified
Walter et al. (2024)	4–7 months; 20 months; 26 months	MuSCs, FAPs, ECs, PCs, Macrophages	1, 2, 3.5, 5 and 7 dpi	Upregulation of "Sen Score" based on scRNA-seq data	Not identified	N.A.	Not identified
Moiseeva et al. (2023)	3–6 months; 28 months	FAPs, Myeloid cells, MuSCs	3 and 7 dpi	SA-β-Gal↑, γH2AX↑, Lamin B1↓, p16^INK4a^↑	Anti-regenerative (in both young and aged mice)	Senescent cell clearance with *p16-3MR* and D + Q enhanced regeneration	Senescent cells create an aged-like, inflamed niche via pro-fibrotic and pro-inflammatory SASP, disrupting stem cell proliferation

### Early evidence: the emergence of a transient pro-regenerative role

The transient emergence of senescent cells in regenerating muscle was detected by several pioneering investigations, primarily using canonical markers like SA-β-Gal activity and the expression of cell-cycle inhibitors like p16^INK4a^. In 2015, Le Roux et al. first demonstrated the induction of senescence in tibialis anterior (TA) muscle under both acute muscle injury by cardiotoxin (CTX) injection in 6–8-week-old mice and chronic muscle injury in 8-week-old *mdx* mice, a Duchenne muscular dystrophy model characterized by persistent inflammation and aberrant fibrosis. This was evidenced by increased SA-β-Gal staining and the absence of the proliferation marker Ki67. More than half of the SA-β-Gal^+^ cells expressed the endothelial marker Flk-1, while only a subset expressed the macrophage marker F4/80 and none showed detectable levels of Pax7, Tcf4, or the pericyte marker Ng2. These SA-β-Gal⁺ senescent cells were absent in homeostatic muscle but accumulated transiently during early stage of regeneration. Notably, the proportion of SA-β-Gal^+^ cells was reduced by approximately 25% in p16^INK4a^/p19^Arf^ mutant mice and no significant differences were detected in p21- or p53-deficient mice, implicating the role of p16^INK4a^ and/or p19^Arf^ in regulation of senescence induction during muscle regeneration (Le Roux et al. 2015). Although this study suggested a potential role for senescent cells in muscle regeneration, it did not experimentally establish their functional contribution.

In a subsequent study, Chiche et al. also reported the emergence of senescent cells in TA muscle of 6–8 weeks old mice at 10 days post injury (dpi) following CTX injury, characterized by markedly higher levels of SA-β-Gal^+^ cells and elevated expression of p16^INK4a^, p19^Arf^, and SASP components. Through the induction of in vivo partial reprogramming, mediated by short-term, doxycycline-controlled expression of the Yamanaka factors (OSKM), the authors observed the transient emergence of Nanog⁺ cells (cell pluripotency and reprogramming marker) during skeletal muscle regeneration, indicating increased cellular plasticity in the regenerating niche. These cells primarily originate from Pax7⁺ MuSCs and are spatially closely associated with injury-induced senescent cells. Mechanistically, SASP factors, particularly IL-6, act non-cell-autonomously to promote this plastic state. This enhanced plasticity significantly improves the regenerative capacity of muscle tissue without progressing to a fully pluripotent state or inducing teratoma formation. The selective elimination of these senescent cells through ABT-263 (which induces senescent cell apoptosis) treatment or in *p16-3MR* mice (which clear p16^INK4a^-positive cells) reduced both senescent cell burden and cellular plasticity during muscle regeneration, indicating senescence is important for cellular plasticity in vivo (Chiche et al. 2017). However, the types of senescent cells responsible for the observed effects were unidentified.

Later investigation by Saito et al. identified FAPs as a predominant cell population at 4 dpi in acute injury induced by barium chloride in gastrocnemius muscle of 8–10-week-old mice. These FAPs were characterized by a classical senescent phenotype, including markedly elevated SA-β-Gal activity, upregulation of p53, p16^INK4a^, p19^Arf^, p21, SASP components and γH2AX foci formation. To investigate the functional significance of these senescent FAPs in muscle regeneration, FAPs isolated from control (p53^+/+^) or p53‑mutant (p53^−/−^) mice were examined. In macrophage–FAP co-culture assay, H_2_O_2_ induced senescent p53^+/+^ FAPs increased macrophage-mediated phagocytic clearance of FAPs, a critical process that prevents their pathological accumulation and subsequent fibrosis. FAP-myoblast co-culture assay showed myoblast differentiation was promoted by co-culture with H_2_O_2_-treated p53^+/+^ FAPs but inhibited by H_2_O_2_-treated p53^−/−^ FAPs. This pro-regenerative role was confirmed in vivo by transplanting senescent p53^+/+^ FAPs into gastrocnemius followed by injury, which led to enhanced regeneration and reduced fibrosis (Saito et al. 2020).

In summary, these early studies identified the transient emergence of senescent cells during muscle regeneration through SA-β-Gal staining and the detection of cell-cycle inhibitor genes and demonstrated that effective muscle regeneration may rely on the transient, localized accumulation of senescent cells.

### New insights from single cell and spatial mapping

With recent advances in sequencing technologies, novel approaches such as single-cell RNA sequencing (scRNA-seq) and spatial transcriptomics have significantly enhanced our ability to identify and characterize senescent cells. Since 2022, multiple studies have leveraged these techniques to unravel the single-cell and spatial landscapes of senescence following muscle injury. Through spatial transcriptomic profiling of CTX-injured gastrocnemius muscles at 2 and 5 dpi in 8-week-old mice, Young et al. demonstrated the regions exhibiting elevated senescence and SASP gene activity were precisely localized to the zones of active tissue repair and inflammation. Complementary scRNA-seq analysis of mononuclear cells from 5 dpi TA muscle revealed a significant upregulation of key senescence pathway markers, including p53, p57^Kip2^, p27, p21, and GLB1, across diverse cell types. Most of them were prominently in FAPs and macrophages, but also in ECs, Tenocytes, and a subset of MuSCs. Subsequently, SA-β-Gal staining assay showed SA‑β‑Gal⁺ cells were absent at 0 and 1 dpi, became detectable at 3 dpi, and remained elevated at 5 dpi. By 21 dpi, the number of SA-β-Gal^+^ cells gradually declined to an undetectable level, confirming the transient nature of senescence during regeneration. In contrast, SA‑β‑Gal staining combined with spatial transcriptomic profiling of gastrocnemius muscles from chronic injured *mdx* mice revealed the persistent accumulation of senescent cells within regenerative areas. To definitively establish the function of these senescent-like cells, the authors utilized the ABT-263 to selectively eliminate them during the regeneration process after the acute muscle injury. This intervention resulted in a significant reduction in SA-β-Gal^+^ cells and impaired muscle repair, characterized by reduction in myofiber cross-sectional area (CSA) and Pax7^+^ MuSC abundance (Young et al. 2021; Young et al. 2022). This study suggests that an acute, transient senescence state in multiple cell types within the muscle niche is not merely a bystander effect but is required for optimal myofiber growth and MuSCs dynamics during regeneration, akin to its role in skin wound healing and neonatal heart repair.

To refine identification, informatics scoring methods based on senescence-related gene expression signatures were applied to scRNA-seq datasets. Among them, Walter et al. developed a one-way Sen Score method, built upon upregulated senescence-signature genes across 6 gene lists. This scoring system was applied to scRNA-seq data from TA muscle of young (4–7 months), old (20 months), and geriatric (26 months) mice at multiple time points following acute notexin-induced injury, as well as to spatial transcriptomic maps from 5 dpi TA muscle of young and geriatric mice. High senescence scores were identified in several cell populations, including MuSCs and progenitors, FAPs, ECs, PCs, and monocytes/macrophages. Later analysis in myogenic cells uncovered a transitory senescent cell population that was abundant at 3.5 dpi across all ages but increased in older mice. These cells were enriched in the self-renewing MuSC stage and associated with impaired regenerative capacity, suggesting that cellular senescence contributes to stalled MuSC function in aging (Walter et al. 2024). Very recently, Yang et al. also created a unified senescence score (USS) by integrating four senescence-associated gene sets to identify senescent cells in human skeletal muscle. Each cell was assigned a score based on its senescence gene expression profile. Using this approach, senescent cells were identified in MuSCs, ECs, smooth muscle cells, and FAPs of young human muscle and significantly increased in aged muscle (Li et al. 2025b). Together, these studies based on single cell and spatial mapping demonstrate the heterogeneity and dynamics of senescent cell populations during muscle regeneration; however, the functional implications of these senescent populations on muscle regeneration were not dissected.

### Contradictory view: the detrimental role of senescence in muscle regeneration

**Fig. 1:**
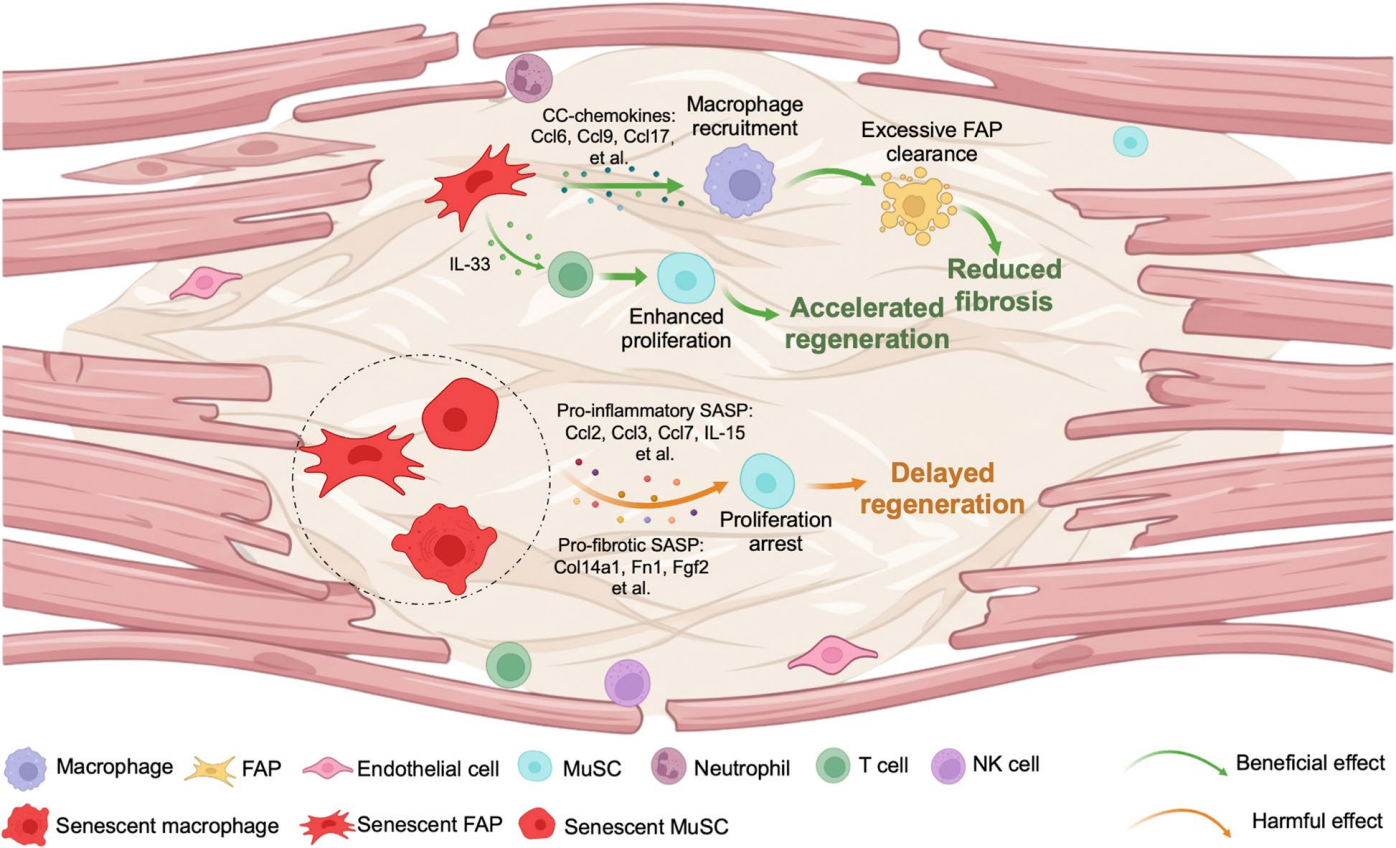
Function of senescent cells in skeletal muscle regeneration. During the injury induced muscle regenerative process, multiple niche cells,including immune cells, FAPs, ECs, and MuSCs, coordinate tissue repair. Senescent cells arising primarily from macrophages, FAPs, and MuSCs accumulate within this niche and secrete a spectrum of SASP factors. Controversial findings exist regarding the beneficial or harmful role of senescent cells

While the above studies have primarily supported the beneficial contribution of senescent cells in muscle regeneration, contradictory findings arose to suggest these cells may impair repair processes (Fig. 1).

Recent study from Dungan et al. illustrated the paradoxical, context-dependent nature of cellular senescence in muscle regeneration. They investigated the role of senescent cells in BaCl_2_ injured TA muscle of both young (3-month-old) and old (20-month-old) mice. The results revealed that SA-β-Gal^+^ cells, predominantly comprising CD11b^+^ macrophages, were scarce in uninjured muscle but increased markedly at 7 dpi in both age groups. At 14 dpi, these cells in aged mice exhibited an elevated expression of senescence and SASP factors genes (e.g., p21, Ccl2, Il1b), whereas in young mice they transitioned toward a reparative M2-macrophages like state as bulk RNA‑seq of the SA‑β‑Gal⁺ cells revealed higher expression of Adamts1 and Pdgfb, factors typically secreted by M2 macrophages. At 28 dpi, SA-β-Gal^+^ cells remained elevated only in old mice, correlating with impaired regeneration. D + Q (dasatinib and quercetin, which target anti‑apoptotic pathways to globally eliminate senescent cells) treatment specifically reduced SA-β-Gal^+^ cell burden and enhanced muscle regeneration in old mice, evidenced by larger myofiber CSA, increased MuSCs abundance, and improved physical function. Conversely, D + Q impaired regeneration in young mice, reducing fiber CSA and MuSCs activation. Besides, D + Q directly promoted myogenic progenitor cell proliferation only in cells from old mice in vitro (Dungan et al. 2022). These findings indicate that senescent cells accumulate persistently in aged muscle post-injury, contributing to regenerative decline, whereas in young muscle they emerge transiently and promote regeneration. Senolytic intervention ameliorates this regenerative deficit in old mice by clearing senescent macrophages and enhancing progenitor cell function, but disrupts regenerative processes in young mice, highlighting the age-specific efficacy of senescent cells.

Interestingly, more recently, Moiseeva et al. argued that senescent cells emerging transiently in acutely injured muscles of both young (2–6 months old) and aged mice (28 months old) exert harmful effects on muscle regeneration. To comprehensively identify and characterize the senescent cells, fluorescence-activated cell sorting (FACS) based on SPiDER-β-gal live-cell staining was employed to isolate senescent and non-senescent cells, which were then profiled by scRNA-seq. This analysis revealed that senescent cells, primarily FAPs, myeloid cells, and MuSCs, exhibit established senescence markers such as persistent DNA damage foci (γH2AX^+^), heightened SA-β-gal activity, larger cell size, loss of lamin B1 and cell cycle arrest. Compared with non-senescent counterparts, senescent MuSCs, FAPs, and myeloid cells of young mice muscle at 3 dpi exhibit largely distinct transcriptional profiles, each enriched in cell type–specific pathways. Persistent accumulation of senescent cells was also observed in chronic injury *mdx* mice. Depleting senescent cells both in acute injured muscle and chronic injury *mdx* mice through genetic clearance models (*p16-3MR* mice) or pharmacological senolytics (D + Q) enhances regenerative outcomes, reduces fibrosis and inflammation, and restores muscle function. Consistently, transplantation of SPiDER^+^ cells but not SPiDER^−^ isolated from 3 dpi muscle into pre-injured muscle at 2 dpi delayed regeneration of young host muscles. Mechanistically, senescent cells create an aged-like, inflamed niche via pro-fibrotic and pro-inflammatory SASPs, which disrupt MuSC proliferation and impede tissue repair (Moiseeva et al. 2023). This study identified specific senescent cell types with distinct transcriptional profiles in regenerating muscle and compellingly demonstrates that these senescent populations negatively affect muscle regeneration in both young and aged mice. However, it remains unclear whether each senescent cell type may exert divergent, even opposing, effects on muscle repair.

## Conclusions and perspectives

Broadly speaking, cellular senescence exerts a beneficial role in tissue regeneration; however, its role in skeletal muscle regeneration remains unresolved. The evidence reviewed herein underscores the complex and paradoxical role of cellular senescence in skeletal muscle regeneration. Most studies indicate that senescence promotes skeletal muscle regeneration, potentially through the transient senescence state and SASP-mediated modulation of the immune response, stem cell activation, and matrix remodeling. Nevertheless, opposing evidence exists to demonstrate the inhibitory effect of senescent cells on muscle regeneration by establishing an inflammatory niche. These conflicting observations underscore the need to address several key gaps before the field can reach a coherent understanding of how senescence shapes muscle repair.

First, a fundamental gap in current studies is the heterogeneity of senescent cells in regenerating muscle. Multiple cell types undergo senescence during muscle regeneration, and their heterogeneity should not be overlooked. Different senescent cell populations have distinct transcriptional profiles and may exert unique or even opposing functions in regeneration. The predominant use of global senescent cell clearance strategies, such as the senolytic drug (ABT-263 and D + Q) and the *p16-3MR* transgenic mouse model, obscures the specific contributions of individual senescent cell populations. These approaches indiscriminately eliminate p16^INK4a^-positive senescent cells across all types. Consequently, the net outcome observed in regeneration likely represents the aggregate effect of eliminating a heterogeneous mixture of senescent cells, whose distinct and potentially opposing functions via cell-type-specific SASPs are conflated. Compounding this issue, the *p16-3MR* model has been demonstrated to exhibit inefficient clearance of senescent cells (Hori et al. 2024), further confounding data interpretation. Therefore, defining each senescent type, characterizing their features and the unique mechanistic roles in muscle regeneration are the next urgent steps. Future research on developing senotherapeutics for enhancing muscle regeneration should therefore account for senescent cell heterogeneity by developing targeted clearance strategies that selectively eliminate deleterious subsets while preserving pro-regenerative ones.

Second, it is crucial to distinguish between the transient senescence induced by acute muscle injury and the persistent senescence associated with chronic muscle pathology, such as in *mdx* or aged mice. The roles of these two forms of senescence may diverge in their influence on muscle regeneration. The transient senescence has been implicated in facilitating key regenerative processes, such as cellular plasticity, phagocytic clearance, and myoblast differentiation. However, the studies reviewed here demonstrated that the clearance of persistent senescent cells in either *mdx* mice or in acute injury induced regeneration of aged mice promotes muscle regeneration, indicating the harmful effects of persistent senescent cells. Thus, future investigations must employ context‑specific models to dissect the distinct molecular signatures, cellular origins, and functional consequences of senescence in acute regenerative versus chronic degenerative environments.

Third, lack of insight into the fate of senescent cells during muscle regeneration is another gap to fill. Most evidence relies on canonical markers such as reduced Ki67, elevated p16^INK4a^/p21 expression and persistent DNA damage foci, which indicate a growth arrest associated state but reveal little about what ultimately happens to these cells. For instance, a recent study showed that the p16^INK4a^-positive ECs could survive and re-enter the cell cycle during liver repair, suggesting that injury‑induced senescence may be reversible for certain cell types (Zhao et al. 2024). Yet current studies in skeletal muscle regeneration rarely determine whether senescent cells persist and are cleared, or instead recover and resume proliferation during later stages of regeneration. Without longitudinal tracking, lineage tracing, or functional reactivation assays, it remains unclear whether the cells identified by canonical markers represent transiently arrested populations that contribute to repair or more persistent senescent cells with distinct consequences. Clarifying senescent‑cell fate will be essential for understanding their mechanistic roles and for avoiding the inadvertent removal of transiently arrested cells that may support tissue repair.

Finally, an important and largely unexplored gap concerns the upstream mechanisms that drive the emergence of senescent cells during muscle regeneration. The signals and pathways that initiate senescence in the regenerating niche remain poorly characterized. Addressing this question is essential for understanding how senescence is regulated in acute versus chronic contexts and for designing interventions that modulate rather than merely eliminate senescent cells.

## Data Availability

Not applicable.

## Abbreviations

SASP: Senescence-associated secretory phenotype
MuSCs: Muscle stem cells
FAPs: Fibro-adipogenic progenitors
ECs: Endothelial cells
DMD: Duchenne muscular dystrophy
QSCs: Quiescent muscle stem cells
M1: Pro-inflammatory polarized macrophage
M2: Anti-inflammatory polarized macrophage
Tregs: Regulatory T cells
ECM: Extracellular matrix
PCs: Pericytes
NMJs: Neuromuscular junctions
AChRs: Acetylcholine receptors
MMPs: Matrix metalloproteinases
mTOR: Mammalian target of rapamycin
SA-β-Gal: Senescence-associated β-galactosidase
TA: Tibialis anterior
CTX: Cardiotoxin
Dpi: Days post injury
scRNA-seq: Single-cell RNA sequencing
CSA: Cross-sectional area
USS: Unified senescence score
FACS: Fluorescence-activated cell sorting
D + Q: Dasatinib and quercetin

## References

[CR1] Ajoolabady A, Pratico D, Bahijri S, Tuomilehto J, Uversky VN, Ren J. Hallmarks of cellular senescence: biology, mechanisms, regulations. Exp Mol Med. 2025;57(7):1482–91. 10.1038/s12276-025-01480-7. 40634753 10.1038/s12276-025-01480-7 PMC12322015

[CR2] Ancel S, Mashinchian O, Feige JN. Adipogenic progenitors keep muscle stem cells young. Aging (Albany NY). 2019;11(18):7331–3. 10.18632/aging.102304. 31537755 10.18632/aging.102304 PMC6782005

[CR3] Antelo-Iglesias L, Picallos-Rabina P, Estévez-Souto V, Da Silva-Álvarez S, Collado M. The role of cellular senescence in tissue repair and regeneration. Mech Ageing Dev. 2021;198:111528. 10.1016/j.mad.2021.111528. 34181964 10.1016/j.mad.2021.111528

[CR4] Baker DJ, Childs BG, Durik M, Wijers ME, Sieben CJ, Zhong J, et al. Naturally occurring p16Ink4a-positive cells shorten healthy lifespan. Nature. 2016;530(7589):184–9. 10.1038/nature16932. 26840489 10.1038/nature16932 PMC4845101

[CR5] Bernard C, Zavoriti A, Pucelle Q, Chazaud B, Gondin J. Role of macrophages during skeletal muscle regeneration and hypertrophy-Implications for immunomodulatory strategies. Physiol Rep. 2022;10(19):e15480. 10.14814/phy2.15480. 36200266 10.14814/phy2.15480 PMC9535344

[CR6] Birch J, Gil J. Senescence and the SASP: many therapeutic avenues. Genes Dev. 2020;34(23–24):1565–76. 10.1101/gad.343129.120. 33262144 10.1101/gad.343129.120 PMC7706700

[CR7] Burzyn D, Kuswanto W, Kolodin D, Shadrach Jennifer L, Cerletti M, Jang Y, et al. A Special Population of Regulatory T Cells Potentiates Muscle Repair. Cell. 2013a;155(6):1282–95. 10.1016/j.cell.2013.10.054. 10.1016/j.cell.2013.10.054 PMC3894749 24315098

[CR8] Burzyn D, Kuswanto W, Kolodin D, Shadrach JL, Cerletti M, Jang Y, et al. A special population of regulatory T cells potentiates muscle repair. Cell. 2013;155(6):1282–95. 10.1016/j.cell.2013.10.054. 24315098 10.1016/j.cell.2013.10.054 PMC3894749

[CR9] Byun WS, Lee J, Baek J-H. Beyond the bulk: overview and novel insights into the dynamics of muscle satellite cells during muscle regeneration. Inflamm Regen. 2024;44(1):39. 10.1186/s41232-024-00354-1. 39327631 10.1186/s41232-024-00354-1 PMC11426090

[CR10] Chang J, Wang Y, Shao L, Laberge R-M, Demaria M, Campisi J, et al. Clearance of senescent cells by ABT263 rejuvenates aged hematopoietic stem cells in mice. Nat Med. 2016;22(1):78–83. 10.1038/nm.4010. 26657143 10.1038/nm.4010 PMC4762215

[CR11] Chazaud B. Inflammation and Skeletal Muscle Regeneration: Leave It to the Macrophages! Trends Immunol. 2020;41(6):481–92. 10.1016/j.it.2020.04.006. 32362490 10.1016/j.it.2020.04.006

[CR12] Chiche A, Le Roux I, von Joest M, Sakai H, Aguín SB, Cazin C, et al. Injury-induced senescence enables in vivo reprogramming in skeletal muscle. Cell Stem Cell. 2017;20(3):407-14.e4. 10.1016/j.stem.2016.11.020. 28017795 10.1016/j.stem.2016.11.020

[CR13] Demaria M, Ohtani N, Youssef SA, Rodier F, Toussaint W, Mitchell JR, et al. An essential role for senescent cells in optimal wound healing through secretion of PDGF-AA. Dev Cell. 2014;31(6):722–33. 10.1016/j.devcel.2014.11.012. 25499914 10.1016/j.devcel.2014.11.012 PMC4349629

[CR14] Di Micco R, Krizhanovsky V, Baker D, d’Adda di Fagagna F. Cellular senescence in ageing: from mechanisms to therapeutic opportunities. Nat Rev Mol Cell Biol. 2021;22(2):75–95. 10.1038/s41580-020-00314-w. 33328614 10.1038/s41580-020-00314-w PMC8344376

[CR15] Duan D, Goemans N, Takeda Si, Mercuri E, Aartsma-Rus A. Duchenne muscular dystrophy. Nature Reviews Disease Primers. 2021;7(1):13. 10.1038/s41572-021-00248-3. 10.1038/s41572-021-00248-3 PMC10557455 33602943

[CR16] Dungan CM, Murach KA, Zdunek CJ, Tang ZJ, Nolt GL, Brightwell CR, et al. Deletion of SA β-Gal+ cells using senolytics improves muscle regeneration in old mice. Aging Cell. 2022;21(1):e13528. 10.1111/acel.13528. 34904366 10.1111/acel.13528 PMC8761017

[CR17] Fang J, Feng C, Chen W, Hou P, Liu Z, Zuo M, et al. Redressing the interactions between stem cells and immune system in tissue regeneration. Biol Direct. 2021;16(1):18. 10.1186/s13062-021-00306-6. 34670590 10.1186/s13062-021-00306-6 PMC8527311

[CR18] Feng LT, Chen ZN, Bian H. Skeletal muscle: molecular structure, myogenesis, biological functions, and diseases. MedComm (2020). 2024;5(7):e649. 10.1002/mco2.649. 38988494 10.1002/mco2.649 PMC11234433

[CR19] Feng T, Meng J, Kou S, Jiang Z, Huang X, Lu Z, et al. CCN1-induced cellular senescence promotes heart regeneration. Circulation. 2019;139(21):2495–8. 10.1161/CIRCULATIONAHA.119.039530. 31107624 10.1161/CIRCULATIONAHA.119.039530

[CR20] Flores-Opazo M, Kopinke D, Helmbacher F, Fernández-Verdejo R, Tuñón-Suárez M, Lynch GS, et al. Fibro-adipogenic progenitors in physiological adipogenesis and intermuscular adipose tissue remodeling. Mol Aspects Med. 2024;97:101277. 10.1016/j.mam.2024.101277. 38788527 10.1016/j.mam.2024.101277 PMC11692456

[CR21] Hayflick L, Moorhead PS. The serial cultivation of human diploid cell strains. Exp Cell Res. 1961;25:585–621. 10.1016/0014-4827(61)90192-6. 13905658 10.1016/0014-4827(61)90192-6

[CR22] Henrot P, Blervaque L, Dupin I, Zysman M, Esteves P, Gouzi F, et al. Cellular interplay in skeletal muscle regeneration and wasting: insights from animal models. J Cachexia Sarcopenia Muscle. 2023;14(2):745–57. 10.1002/jcsm.13103. 36811134 10.1002/jcsm.13103 PMC10067506

[CR23] Heredia JE, Mukundan L, Chen FM, Mueller AA, Deo RC, Locksley RM, et al. Type 2 innate signals stimulate fibro/adipogenic progenitors to facilitate muscle regeneration. Cell. 2013;153(2):376–88. 10.1016/j.cell.2013.02.053. 23582327 10.1016/j.cell.2013.02.053 PMC3663598

[CR24] Herranz N, Gil J. Mechanisms and functions of cellular senescence. J Clin Invest. 2018;128(4):1238–46. 10.1172/jci95148. 29608137 10.1172/JCI95148 PMC5873888

[CR25] Hori N, Kawamoto S, Uemura K, Okumura Y, Tanaka K, Park JH, et al. Shaving black fur uncovers hidden issues in p16–3MR mice. bioRxiv. 2024:2024.06.24.600181. 10.1101/2024.06.24.600181.

[CR26] Huang W, Hickson LJ, Eirin A, Kirkland JL, Lerman LO. Cellular senescence: the good, the bad and the unknown. Nat Rev Nephrol. 2022;18(10):611–27. 10.1038/s41581-022-00601-z. 35922662 10.1038/s41581-022-00601-z PMC9362342

[CR27] Jun J-I, Lau LF. The matricellular protein CCN1 induces fibroblast senescence and restricts fibrosis in cutaneous wound healing. Nat Cell Biol. 2010;12(7):676–85. 10.1038/ncb2070. 20526329 10.1038/ncb2070 PMC2919364

[CR28] Kedlian VR, Wang Y, Liu T, Chen X, Bolt L, Tudor C, et al. Human skeletal muscle aging atlas. Nat Aging. 2024;4(5):727–44. 10.1038/s43587-024-00613-3. 38622407 10.1038/s43587-024-00613-3 PMC11108788

[CR29] Kharraz Y, Guerra J, Mann CJ, Serrano AL, Muñoz-Cánoves P. Macrophage plasticity and the role of inflammation in skeletal muscle repair. Mediators Inflamm. 2013;2013:491497. 10.1155/2013/491497. 23509419 10.1155/2013/491497 PMC3572642

[CR30] Kim K-H, Chen C-C, Monzon RI, Lau LF. Matricellular protein CCN1 promotes regression of liver fibrosis through induction of cellular senescence in hepatic myofibroblasts. Mol Cell Biol. 2013;33(10):2078–90. 10.1128/MCB.00049-13. 23508104 10.1128/MCB.00049-13 PMC3647960

[CR31] Koike H, Manabe I, Oishi Y. Mechanisms of cooperative cell-cell interactions in skeletal muscle regeneration. Inflamm Regen. 2022;42(1):48. 10.1186/s41232-022-00234-6. 36380396 10.1186/s41232-022-00234-6 PMC9667595

[CR32] Krizhanovsky V, Yon M, Dickins RA, Hearn S, Simon J, Miething C, et al. Senescence of activated stellate cells limits liver fibrosis. Cell. 2008;134(4):657–67. 10.1016/j.cell.2008.06.049. 18724938 10.1016/j.cell.2008.06.049 PMC3073300

[CR33] Lavarti R, Alvarez-Diaz T, Marti K, Kar P, Raju RP. The context-dependent effect of cellular senescence: from embryogenesis and wound healing to aging. Ageing Res Rev. 2025;109:102760. 10.1016/j.arr.2025.102760. 40318767 10.1016/j.arr.2025.102760 PMC12145239

[CR34] Le Roux I, Konge J, Le Cam L, Flamant P, Tajbakhsh S. Numb is required to prevent p53-dependent senescence following skeletal muscle injury. Nat Commun. 2015;6(1):8528. 10.1038/ncomms9528. 26503169 10.1038/ncomms9528 PMC4639798

[CR35] Li W, Chen M, Zhang L. Muscle stem cell microenvironment and functions in muscle regeneration. Biomolecules. 2025a;15(6):765. 40563407 10.3390/biom15060765 PMC12190381

[CR36] Li Y, Li C, Zhou Q, Liu X, Qiao Y, Xie T, et al. Multiomics and cellular senescence profiling of aging human skeletal muscle uncovers Maraviroc as a senotherapeutic approach for sarcopenia. Nat Commun. 2025b;16(1):6207. 10.1038/s41467-025-61403-y. 40617829 10.1038/s41467-025-61403-y PMC12228793

[CR37] Li Y, Wang H. Cellular Senescence in Skeletal Muscle Aging. J Korean Endocr Soc. 2026;0. 10.3803/EnM.2025.2816. 10.3803/EnM.2025.2816 PMC13172641 41833287

[CR38] Loomis T, Smith LR. Thrown for a loop: fibro-adipogenic progenitors in skeletal muscle fibrosis. Am J Physiol Cell Physiol. 2023;325(4):C895-c906. 10.1152/ajpcell.00245.2023. 37602412 10.1152/ajpcell.00245.2023 PMC11932532

[CR39] López-Otín C, Blasco MA, Partridge L, Serrano M, Kroemer G. Hallmarks of aging: an expanding universe. Cell. 2023;186(2):243–78. 10.1016/j.cell.2022.11.001. 36599349 10.1016/j.cell.2022.11.001

[CR40] Lucas V, Cavadas C, Aveleira CA. Cellular senescence: from mechanisms to current biomarkers and senotherapies. Pharmacol Rev. 2023;75(4):675–713. 10.1124/pharmrev.122.000622. 36732079 10.1124/pharmrev.122.000622

[CR41] Luo YE, Abe-Teh Z, Alsaghir TY, Kuo LY, Yu F, Stoker BE, et al. Fibro-Adipogenic Progenitors require autocrine IGF-I in homeostatic and regenerating skeletal muscle. bioRxiv. 2025. 10.1101/2025.04.11.648330. 10.1016/j.stemcr.2026.102973 PMC13385424 42349420

[CR42] Martínez-Zamudio RI, Roux P-F, de Freitas JANLF, Robinson L, Doré G, Sun B, et al. AP-1 imprints a reversible transcriptional programme of senescent cells. Nat Cell Biol. 2020;22(7):842–55. 10.1038/s41556-020-0529-5. 32514071 10.1038/s41556-020-0529-5 PMC7899185

[CR43] Moiseeva V, Cisneros A, Sica V, Deryagin O, Lai Y, Jung S, et al. Senescence atlas reveals an aged-like inflamed niche that blunts muscle regeneration. Nature. 2023;613(7942):169–78. 10.1038/s41586-022-05535-x. 36544018 10.1038/s41586-022-05535-x PMC9812788

[CR44] Patsalos A, Halasz L, Medina-Serpas MA, Berger WK, Daniel B, Tzerpos P, et al. A growth factor–expressing macrophage subpopulation orchestrates regenerative inflammation via GDF-15. J Exp Med. 2021. 10.1084/jem.20210420. 34846534 10.1084/jem.20210420 PMC8635277

[CR45] Rodríguez C, Timóteo-Ferreira F, Minchiotti G, Brunelli S, Guardiola O. Cellular interactions and microenvironment dynamics in skeletal muscle regeneration and disease. Front Cell Dev Biol. 2024;12:1385399. 10.3389/fcell.2024.1385399. 38840849 10.3389/fcell.2024.1385399 PMC11150574

[CR46] Rossi M, Abdelmohsen K. The emergence of senescent surface biomarkers as senotherapeutic targets. Cells. 2021. 10.3390/cells10071740. 34359910 10.3390/cells10071740 PMC8305747

[CR47] Saini J, McPhee JS, Al-Dabbagh S, Stewart CE, Al-Shanti N. Regenerative function of immune system: modulation of muscle stem cells. Ageing Res Rev. 2016;27:67–76. 10.1016/j.arr.2016.03.006. 27039885 10.1016/j.arr.2016.03.006

[CR48] Saito Y, Chikenji TS, Matsumura T, Nakano M, Fujimiya M. Exercise enhances skeletal muscle regeneration by promoting senescence in fibro-adipogenic progenitors. Nat Commun. 2020;11(1):889. 10.1038/s41467-020-14734-x. 32060352 10.1038/s41467-020-14734-x PMC7021787

[CR49] Saito Y, Yamamoto S, Chikenji TS. Role of cellular senescence in inflammation and regeneration. Inflamm Regen. 2024;44(1):28. 10.1186/s41232-024-00342-5. 38831382 10.1186/s41232-024-00342-5 PMC11145896

[CR50] Sarig R, Rimmer R, Bassat E, Zhang L, Umansky KB, Lendengolts D, et al. Transient p53-mediated regenerative senescence in the injured heart. Circulation. 2019;139(21):2491–4. 10.1161/CIRCULATIONAHA.119.040125. 31107623 10.1161/CIRCULATIONAHA.119.040125

[CR51] Schafer MJ, Zhang X, Kumar A, Atkinson EJ, Zhu Y, Jachim S, et al. The senescence-associated secretome as an indicator of age and medical risk. JCI Insight. 2020. 10.1172/jci.insight.133668. 32554926 10.1172/jci.insight.133668 PMC7406245

[CR52] Suryadevara V, Hudgins AD, Rajesh A, Pappalardo A, Karpova A, Dey AK, et al. SenNet recommendations for detecting senescent cells in different tissues. Nat Rev Mol Cell Biol. 2024;25(12):1001–23. 10.1038/s41580-024-00738-8. 38831121 10.1038/s41580-024-00738-8 PMC11578798

[CR53] Tedesco FS, Dellavalle A, Diaz-Manera J, Messina G, Cossu G. Repairing skeletal muscle: regenerative potential of skeletal muscle stem cells. J Clin Invest. 2010;120(1):11–9. 10.1172/jci40373. 20051632 10.1172/JCI40373 PMC2798695

[CR54] Walter LD, Orton JL, Ntekas I, Fong EHH, Maymi VI, Rudd BD, et al. Transcriptomic analysis of skeletal muscle regeneration across mouse lifespan identifies altered stem cell states. Nat Aging. 2024;4(12):1862–81. 10.1038/s43587-024-00756-3. 39578558 10.1038/s43587-024-00756-3 PMC11645289

[CR55] Wan M, Gray-Gaillard EF, Elisseeff JH. Cellular senescence in musculoskeletal homeostasis, diseases, and regeneration. Bone Res. 2021;9(1):41. 10.1038/s41413-021-00164-y. 34508069 10.1038/s41413-021-00164-y PMC8433460

[CR56] Xu M, Pirtskhalava T, Farr JN, Weigand BM, Palmer AK, Weivoda MM, et al. Senolytics improve physical function and increase lifespan in old age. Nat Med. 2018;24(8):1246–56. 10.1038/s41591-018-0092-9. 29988130 10.1038/s41591-018-0092-9 PMC6082705

[CR57] Yadav A, Dabur R. Skeletal muscle atrophy after sciatic nerve damage: mechanistic insights. Eur J Pharmacol. 2024;970:176506. 10.1016/j.ejphar.2024.176506. 38492879 10.1016/j.ejphar.2024.176506

[CR58] Yamakawa H, Kusumoto D, Hashimoto H, Yuasa S. Stem cell aging in skeletal muscle regeneration and disease. Int J Mol Sci. 2020;21(5):1830. 32155842 10.3390/ijms21051830 PMC7084237

[CR59] Yang W, Hu P. Skeletal muscle regeneration is modulated by inflammation. J Orthop Translat. 2018;13:25–32. 10.1016/j.jot.2018.01.002. 29662788 10.1016/j.jot.2018.01.002 PMC5892385

[CR60] Young LV, Morrison W, Campbell C, Moore EC, Arsenault MG, Dial AG, et al. Loss of dystrophin expression in skeletal muscle is associated with senescence of macrophages and endothelial cells. Am J Physiol-Cell Physiol. 2021;321(1):C94–103. 10.1152/ajpcell.00397.2020. 33979211 10.1152/ajpcell.00397.2020

[CR61] Young LV, Wakelin G, Cameron AWR, Springer SA, Ross JP, Wolters G, et al. Muscle injury induces a transient senescence-like state that is required for myofiber growth during muscle regeneration. FASEB J. 2022;36(11):e22587. 10.1096/fj.202200289RR. 36190443 10.1096/fj.202200289RR

[CR62] Yu D, Cai Z, Li D, Zhang Y, He M, Yang Y, et al. Myogenic differentiation of stem cells for skeletal muscle regeneration. Stem Cells Int. 2021;2021:8884283. 10.1155/2021/8884283. 33628275 10.1155/2021/8884283 PMC7884123

[CR63] Zeng Q, Gong Y, Zhu N, Shi Y, Zhang C, Qin L. Lipids and lipid metabolism in cellular senescence: emerging targets for age-related diseases. Ageing Res Rev. 2024;97:102294. 10.1016/j.arr.2024.102294. 38583577 10.1016/j.arr.2024.102294

[CR64] Zhang L, Elkahal J, Wang T, Rimmer R, Genzelinakh A, Bassat E, et al. Egr1 regulates regenerative senescence and cardiac repair. Nat Cardiovasc Res. 2024;3(8):915–32. 10.1038/s44161-024-00493-1. 39196027 10.1038/s44161-024-00493-1

[CR65] Zhang X, Habiballa L, Aversa Z, Ng YE, Sakamoto AE, Englund DA, et al. Characterization of cellular senescence in aging skeletal muscle. Nat Aging. 2022;2(7):601–15. 10.1038/s43587-022-00250-8. 36147777 10.1038/s43587-022-00250-8 PMC9491365

[CR66] Zhao H, Liu Z, Chen H, Han M, Zhang M, Liu K, et al. Identifying specific functional roles for senescence across cell types. Cell. 2024;187(25):7314-34.e21. 10.1016/j.cell.2024.09.021. 39368477 10.1016/j.cell.2024.09.021

[CR67] Ziemkiewicz N, Hilliard G, Pullen NA, Garg K. The role of innate and adaptive immune cells in skeletal muscle regeneration. Int J Mol Sci. 2021. 10.3390/ijms22063265. 33806895 10.3390/ijms22063265 PMC8005179

